# Pioglitazone mitigates early brain injury by suppressing neuroinflammation and oxidative stress after subarachnoid hemorrhage: a rodent model study

**DOI:** 10.1080/07853890.2026.2670780

**Published:** 2026-05-12

**Authors:** Kadir Cetinkaya, Mehmet Özgür Özates, Hümeyra Kullukçu, Yasar Ünsal, Atilla Kazancı, Oktay Gürcan, Evrim Önder, Tuba Saadet Deveci Bulut, Ahmet Gürhan Gürcay

**Affiliations:** ^a^ Neurosurgery Department, Hıtıt Unıversıty Corum Erol Olcok Training and Research Hospital, Corum, Türkiye; ^b^ Neurosurgery Department, Yıldırım Beyazıt Unıversıty, Ankara, Türkiye; ^c^ Neurosurgery Department, Mersin Silifke State Hospıtal, Mersin, Türkiye; ^d^ TC Saglik Bakanligi Ankara Sehir Hastanesi, Ankara, Türkiye; ^e^ Pathology Department, Ankara Etlik City Hospital, Ankara, Türkiye; ^f^ Department of Biochemistry, Gazi University Faculty of Medicine, Ankara, Türkiye

**Keywords:** Experimental subarachnoid haemorrhage, early brain injury, pioglitazone, PPAR-γ activation, Neuroinflammation, oxidative stress

## Abstract

**Objective:**

Subarachnoid haemorrhage (SAH) is a devastating neurovascular emergency where outcomes are largely driven by early brain injury (EBI). Neuroinflammation and oxidative stress are central to EBI pathogenesis. This study experimentally evaluated whether pioglitazone attenuates inflammatory and oxidative responses in the early phase after SAH.

**Materials and Methods:**

Thirty-two adult male Sprague–Dawley rats were randomly allocated to four groups (*n* = 8/group): Sham, SAH, SAH+pioglitazone (PIO), and SAH+vehicle (VEH). SAH was induced by autologous blood injection into the cisterna magna. Pioglitazone (10 mg/kg, intraperitoneally) was administered 1 h post-SAH. Serum interleukin-6 (IL-6), interleukin-1β (IL-1β), and malondialdehyde (MDA) levels were quantified by Enzyme-Linked Immunosorbent Assay (ELISA) at 24 h. Hippocampal neuronal injury was assessed using semi-quantitative histopathological analysis of haematoxylin–eosin–stained sections. Acute-phase biochemical and histopathological effects were evaluated; long-term functional outcomes were not assessed.

**Results:**

IL-6, IL-1β, and MDA levels were significantly elevated in all SAH groups compared with sham (*p* < 0.001). Pioglitazone significantly reduced IL-6 compared with SAH (*p* = 0.032) and VEH (*p* = 0.049), though levels remained higher than sham. Conversely, IL-1β and MDA levels in the PIO group were significantly lower than in SAH and VEH (*p* = 0.001 and *p* = 0.002, respectively) and were comparable to sham. Histopathological analysis demonstrated significantly lower hippocampal neuronal degeneration scores in the pioglitazone-treated group (*p* < 0.05).

**Conclusion:**

Early post-SAH administration of pioglitazone suppresses systemic inflammation and lipid peroxidation while mitigating hippocampal neuronal injury. These findings support a potential neuroprotective role of pioglitazone in EBI after SAH, likely mediated *via* peroxisome proliferator-activated receptor-γ (PPAR-γ) activation.

## Introduction

Subarachnoid haemorrhage (SAH) is an acute neurovascular emergency associated with high mortality and morbidity, most commonly resulting from rupture of an intracranial aneurysm [1]. One of the principal determinants of clinical outcome after SAH is early brain injury (EBI), which develops within the first 72 h following haemorrhage [2]. During this period, the abrupt increase in intracranial pressure and the concomitant reduction in cerebral perfusion pressure lead to transient global ischaemia, disruption of cellular energy metabolism, and ultimately widespread neurological dysfunction [3].

The pathophysiology of EBI is multifactorial and highly dynamic. Disruption of blood–brain barrier integrity, cerebral edoema, microcirculatory dysfunction, neuroinflammation, and oxidative stress constitute the major components of this process [4]. Collectively, these pathological mechanisms promote progressive tissue injury by activating multiple cell death pathways in neuronal and glial populations, thereby significantly worsening long-term neurological outcomes [5].

Neuroinflammation plays a pivotal role in the development of EBI after SAH. Blood and haemoglobin degradation products released into the subarachnoid space activate inflammatory signalling pathways in cerebral endothelial cells and drive microglia towards a proinflammatory phenotype [6]. This response enhances the release of proinflammatory cytokines, including interleukin-1β (IL-1β), interleukin-6 (IL-6), and TNF-α, further disrupting blood–brain barrier integrity and exacerbating secondary neuronal injury [6].

Oxidative stress represents another fundamental determinant in the pathogenesis of EBI. Haemoglobin degradation, iron release, and reperfusion processes following SAH lead to a marked increase in reactive oxygen species (ROS) production [7]. Elevated ROS levels promote neuronal dysfunction by inducing oxidative damage to cellular lipids, proteins, and nucleic acids. Increased Malondialdehyde (MDA) levels, a stable biochemical marker of lipid peroxidation, are widely accepted as a quantitative indicator of this oxidative injury [8]. The bidirectional interaction between inflammation and oxidative stress establishes a pathophysiological vicious cycle that drives the progression of EBI [9].

In recent years, ferroptosis—an iron-dependent form of regulated cell death—has emerged as a key contributor to EBI following SAH [10]. This process, characterised by excessive lipid peroxidation, particularly promotes neuronal cell loss and is considered a critical mechanism underlying poor clinical outcomes [11,12]. Given that pioglitazone has been shown to modulate lipid metabolism and oxidative pathways, its potential role in mitigating ferroptosis-related injury warrants investigation.

Peroxisome proliferator-activated receptor-γ (PPAR-γ) is a ligand-dependent nuclear transcription factor that regulates inflammation, oxidative stress, and cell survival pathways [13]. Activation of PPAR-γ has been shown to suppress NF-κB–mediated proinflammatory signalling, enhance antioxidant defense mechanisms, and modulate cellular injury responses. Notably, functional crosstalk between the PPAR-γ and Nrf2 signalling pathways plays a critical role in the regulation of oxidative stress responses [14]. Experimental studies consistently demonstrate that PPAR-γ activation attenuates inflammatory responses and modulates oxidative stress–related cellular processes [14,15]. Accordingly, PPAR-γ agonists have emerged as promising neuroprotective candidates in the pathogenesis of EBI.

PPAR-γ agonists have attracted considerable attention as therapeutic agents with neuroprotective potential based on these biological properties [16]. Experimental evidence indicates that PPAR-γ activation reduces proinflammatory cytokine production, modulates microglial activation, preserves blood–brain barrier integrity, and attenuates oxidative stress markers [17]. Pioglitazone, a clinically established PPAR-γ agonist, has demonstrated anti-inflammatory and antioxidant effects across various models of central nervous system injury [18].

However, the effects of pioglitazone on early brain injury after SAH and the extent to which these effects are reflected in inflammatory and oxidative stress markers, remain insufficiently characterised. In the current literature, the neuroprotective potential of pioglitazone has largely been evaluated using general inflammatory and oxidative parameters, while its biochemical and histopathological impact specifically within the context of EBI has been investigated in only a limited number of studies.

In this study, we aimed to experimentally evaluate the effects of pioglitazone on inflammatory response and oxidative stress during the early post-SAH period by assessing serum IL-1β, IL-6, and MDA levels together with hippocampal histopathological changes. We hypothesised that pioglitazone administration would attenuate inflammatory and oxidative injury in EBI. The findings of this study are expected to provide translational insight into the potential neuroprotective role of pioglitazone in EBI and to inform future therapeutic strategies.

## Materials and methods

### EthicsQQQ statement

The study protocol was reviewed and approved by the Institutional Animal Care and Use Committee (IACUC) of Kobay Experimental Animals Research Laboratory (Approval No: 403). All procedures were performed in accordance with the National Institutes of Health (NIH) Guide for the Care and Use of Laboratory Animals and complied with the ARRIVE guidelines. To minimise animal suffering, all surgical procedures were performed under general anaesthesia (ketamine 60 mg/kg and xylazine 5 mg/kg, i.p.), and cefazolin sodium (50 mg/kg) was administered for prophylaxis. Animals were euthanized by decapitation under deep anaesthesia at the end of the 24-hour experimental period.

### Experimental animals and study design

A total of 32 healthy adult male Sprague–Dawley rats (250–300 g), obtained from Kobay Experimental Animals Inc. (Ankara, Turkey), were used in this study. Animals were housed under standard laboratory conditions at a controlled ambient temperature (23 ± 2 °C) with a 12-hour light/12-hour dark cycle (lights on at 07:00) and had ad libitum access to food and water.

Rats were randomly allocated into four groups (*n* = 8 per group) using a computer-generated randomisation table:

- **Sham group (SH):** Negative control group undergoing cisterna magna puncture without blood injection.
- **Subarachnoid haemorrhage group (SAH):** Positive control group in which experimental SAH was induced without pharmacological treatment.
- **SAH + Pioglitazone group (PIO):** Treatment group receiving pioglitazone after SAH induction.
- **SAH + Vehicle group (VEH):** Control group receiving vehicle only after SAH induction.

The total sample size was 32 rats.

### Exclusion criteria

During the course of the study, a total of four rats were excluded based on predefined criteria to ensure the robustness and validity of the experimental outcomes. One rat was excluded due to insufficient SAH grade observed during post-mortem autopsy, indicating a failure in successful SAH induction. Another rat was excluded due to the development of a severe post-operative infection, characterised by purulent discharge at the surgical site and systemic signs of distress, which necessitated its humane euthanasia. Furthermore, two rats were excluded due to intraoperative mortality resulting from cardiac arrest during the anaesthesia induction phase, prior to the SAH induction procedure. These exclusions were meticulously documented to maintain transparency and adhere to ethical guidelines for animal research.

### Anaesthesia and perioperative monitoring

Cefazolin sodium (50 mg/kg, intraperitoneally) was administered to all animals 30 min before anaesthesia induction for prophylaxis. General anaesthesia was induced with ketamine hydrochloride (60 mg/kg, intraperitoneally; Ketalar^®^, Pfizer, Istanbul, Turkey) combined with xylazine hydrochloride (5 mg/kg, intraperitoneally; Rompun^®^, Bayer, Istanbul, Turkey). Heart rate, respiratory pattern, and rectal body temperature were continuously monitored throughout the surgical procedure. Body temperature was maintained at 37 ± 0.5 °C using a thermostat-controlled heating pad. Although invasive blood pressure monitoring was not performed, the stability of heart rate and respiratory patterns suggested hemodynamic stability throughout the procedure.

### Experimental subarachnoid hemorrhage model

The cisternal SAH model was established according to previously described standard experimental methods [19]. Under general anaesthesia, animals were placed in the prone position. The occipital region was shaved and disinfected with povidone–iodine solution. Following a midline occipital incision, the paravertebral muscles were dissected and the cisterna magna was carefully exposed.

In the sham group, cisterna magna puncture was performed without blood injection. In the SAH, SAH + pioglitazone (PIO), and SAH + vehicle (VEH) groups, 0.3 mL of autologous blood obtained from the tail vein was slowly injected into the cisterna magna. After injection, the needle was kept in place for ∼2 min to prevent blood reflux [19]. The surgical field was then closed in anatomical layers.

### Drug administration

In the pioglitazone treatment group, pioglitazone hydrochloride (Actos^®^, Takeda Pharmaceutical Company, Osaka, Japan) was administered as a single intraperitoneal dose of 10 mg/kg at 1 h after induction of SAH. Prior to administration, pioglitazone was dissolved in polyethylene glycol (PEG) [20].

The timing of drug administration was selected to target the EBI phase after SAH. The 10 mg/kg dose was chosen based on previous literature demonstrating its optimal blood-brain barrier permeability and therapeutic efficacy in acute CNS injury models without significant systemic side effects [20,21].

In the vehicle group, an equivalent volume of PEG was administered intraperitoneally at the same time point using the same route.

### Postoperative care and sacrifice

Following surgery, all animals were returned to individual cages and monitored regularly for general condition, motor activity, and neurological status. Neurological assessments were performed qualitatively to ensure recovery from anaesthesia and to monitor for gross motor deficits. Supportive care was provided under standard laboratory conditions and nutritional and hydration status were checked daily.

All animals were euthanized by decapitation under deep anaesthesia at 24 h after SAH induction. Immediately thereafter, brain tissues were rapidly harvested and processed under appropriate conditions for biochemical and histopathological analyses. The sacrifice time point was selected to evaluate the EBI phase after SAH.

### Biochemical analyses

Prior to sacrifice, blood samples were collected *via* cardiac puncture or tail vein to evaluate systemic biochemical changes following subarachnoid haemorrhage. Samples were allowed to clot at room temperature and then centrifuged at 3000 rpm for 15 min at 4 °C to obtain serum. The serum was aliquoted and stored at −80 °C until analysis. Systemic inflammatory response and oxidative stress were assessed by measuring serum IL-6, IL-1β and MDA levels using commercially available sandwich ELISA kits according to the manufacturers’ instructions. Absorbance was measured at 450 nm using a microplate reader (Thermo Scientific), and concentrations were calculated based on standard calibration curves. All biochemical analyses were performed by laboratory personnel blinded to the experimental groups. The link between the codes and the experimental groups was not revealed until the entire scoring process and biochemical analyses were finalised and documented. All analyses were conducted in the laboratories of the Department of Biochemistry, Gazi University Faculty of Medicine.

### Haematoxylin and eosin staining and histopathological evaluation

Brain tissue samples obtained after sacrifice were fixed in 10% neutral buffered formalin for 24–48 h to ensure structural preservation. Following fixation, tissues were dehydrated through a graded series of ethanol, cleared in xylene, and embedded in paraffin wax. Serial coronal sections were cut at a thickness of 4 µm using a rotary microtome. The Haematoxylin and Eosin (H&E) staining was performed to visualise general morphological changes. Briefly, sections were deparaffinized and rehydrated through descending grades of alcohol to distilled water. The slides were stained with haematoxylin, rinsed in running tap water, and differentiated in an acid-ethanol solution. After blueing the nuclei with lithium carbonate, the sections were counterstained with an eosin-phloxine solution to visualise the cytoplasm. Finally, the slides were dehydrated, cleared, and mounted for microscopic examination using an Olympus IX71 microscope.

### Blinding and semi-quantitative scoring

To eliminate observer bias and maintain the scientific integrity of the results, a strict blinding procedure was followed. All brain tissue slides were assigned unique, non-sequential codes by an independent laboratory assistant who was not involved in the histopathological scoring. Consequently, the experienced neuropathologist at the Pathology Clinic of Etlik City Hospital conducted the evaluations without knowledge of the experimental groups (e.g. Sham, SAH or treatment). The link between the codes and the experimental groups was not revealed until the entire scoring process was finalised and documented.

Histopathological evaluation was conducted in five random, non-overlapping fields per section at 200× and 400× magnifications. Neuronal injury was identified based on characteristic cytomorphological markers: hypereosinophilia (cytoplasmic eosinophilia), shrunken cytoplasm (neuronal shrinkage), and nuclear pyknosis [22]. The severity of the damage was assessed using the following semi-quantitative scoring system:

- **Grade 0:** Normal histological appearance with intact neurons.
- **Grade 1:** Mild neuronal degeneration.
- **Grade 2:** Moderate neuronal degeneration.
- **Grade 3:** Severe neuronal degeneration.

### Statistical analysis

All statistical analyses were performed using SPSS version 22.0 (IBM Corp., Armonk, NY, USA). Continuous variables with normal distribution are presented as mean ± standard deviation, whereas non-normally distributed data are presented as median (minimum–maximum). Normality was assessed using the Shapiro–Wilk test.

For normally distributed variables, intergroup comparisons were performed using one-way analysis of variance (ANOVA), followed by Tukey’s post hoc test when appropriate. For variables that did not meet normality assumptions, the Kruskal–Wallis test was applied; when overall significance was detected, pairwise comparisons were conducted using the Mann–Whitney *U* test.

Each experimental group included eight rats. This sample size was determined a priori by power analysis to detect significant differences in hippocampal neuronal degeneration scores with a significance level of *α* = 0.05 and statistical power of 80%. A two-tailed *p* value < 0.05 was considered statistically significant.

## Results

### Serum IL-6 levels

**Figure 1.**
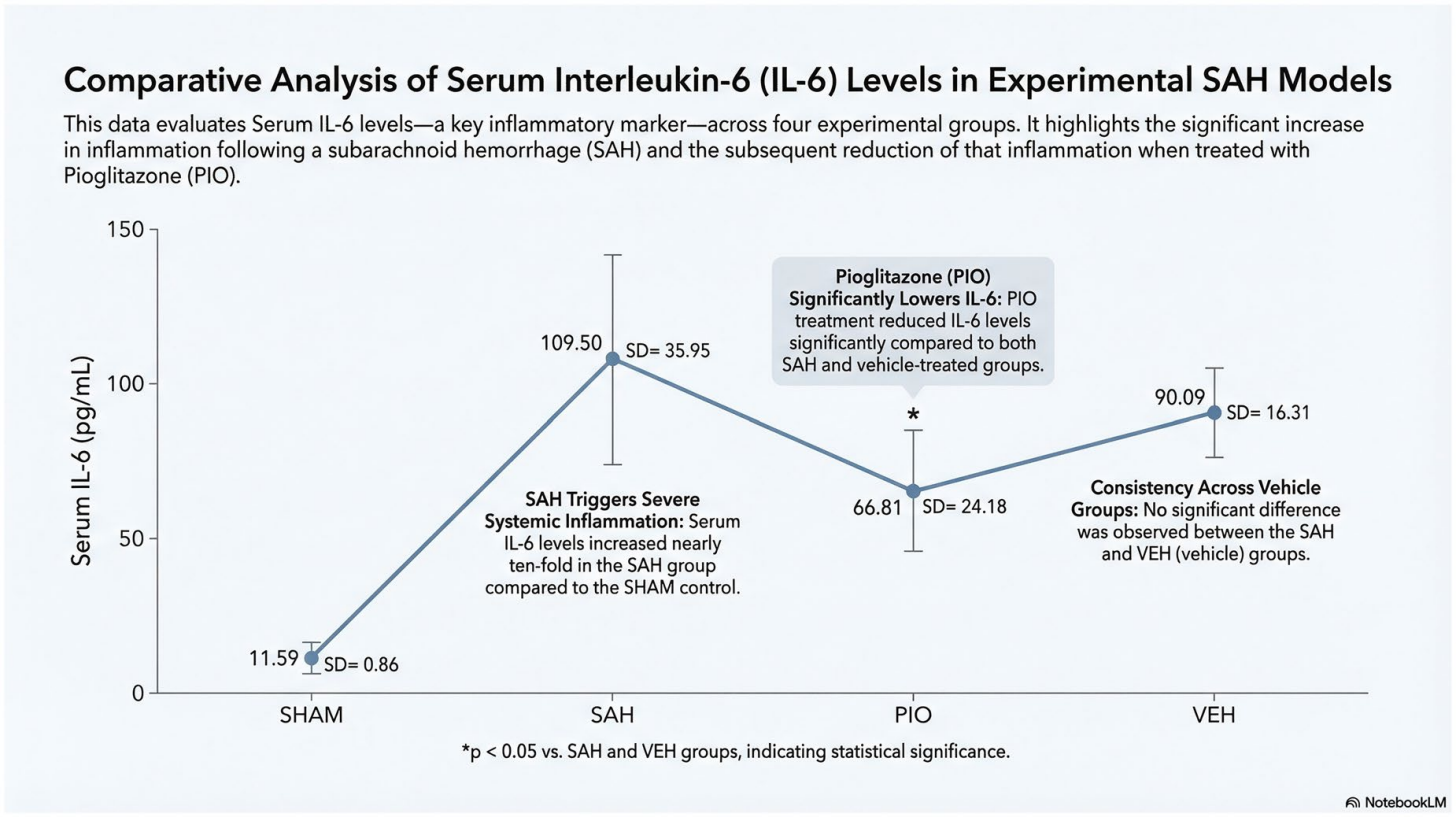
Serum IL-6 levels in experimental groups. Data are presented as mean ± SD; *p* < 0.05 was considered statistically significant. Pioglitazone significantly reduced IL-6 compared with SAH (*p* = 0.032).

Serum IL-6 levels were significantly elevated in all SAH groups compared with the sham group (*p* < 0.001, Figure 1). In the SAH group, the mean IL-6 level was 109.50 ± 35.95 pg/mL. Pioglitazone treatment significantly reduced IL-6 levels to 66.81 ± 24.18 pg/mL compared with the SAH group (*p* = 0.032, Figure 1). IL-6 levels in the PIO group were also significantly lower than those in the VEH group (*p* = 0.049). No significant difference was observed between the SAH and VEH groups (*p* = 0.391). Despite the reduction, IL-6 levels in the pioglitazone group remained significantly higher than in the sham group (*p* < 0.001).

### Serum malondialdehyde (MDA) levels

**Figure 2.**
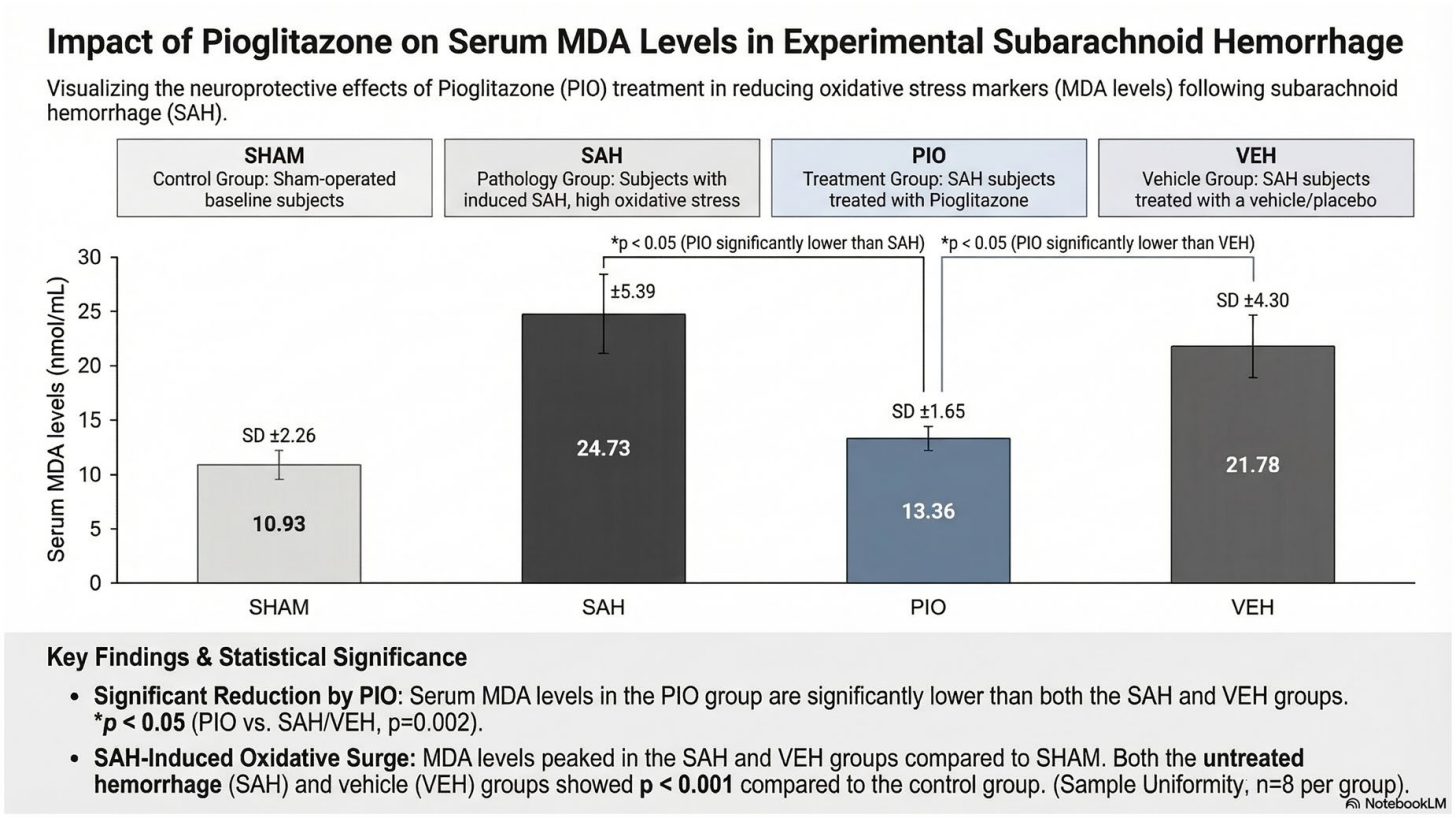
Comparison of serum MDA levels across experimental groups. Data are presented as mean ± SD; *p* < 0.05 was considered statistically significant. MDA levels in the PIO group were significantly lower than in SAH and VEH (*p* = 0.001 and *p* = 0.002, respectively).

Serum MDA levels were significantly elevated in all SAH-induced groups compared with the sham group (*p* < 0.001, Figure 2). In the SAH group, the mean MDA level was 24.73 ± 5.39 nmol/mL. Pioglitazone treatment significantly reduced MDA levels to 13.36 ± 1.65 nmol/mL compared with the SAH group (*p* = 0.002). MDA levels in the PIO group were also significantly lower than those in the VEH group (*p* = 0.002). No significant difference was observed between the SAH and VEH groups (*p* = 0.121). Notably, MDA levels in the pioglitazone group were comparable to those in the sham group, with no significant difference detected (*p* = 0.289).

### Serum IL-1β levels

**Figure 3.**
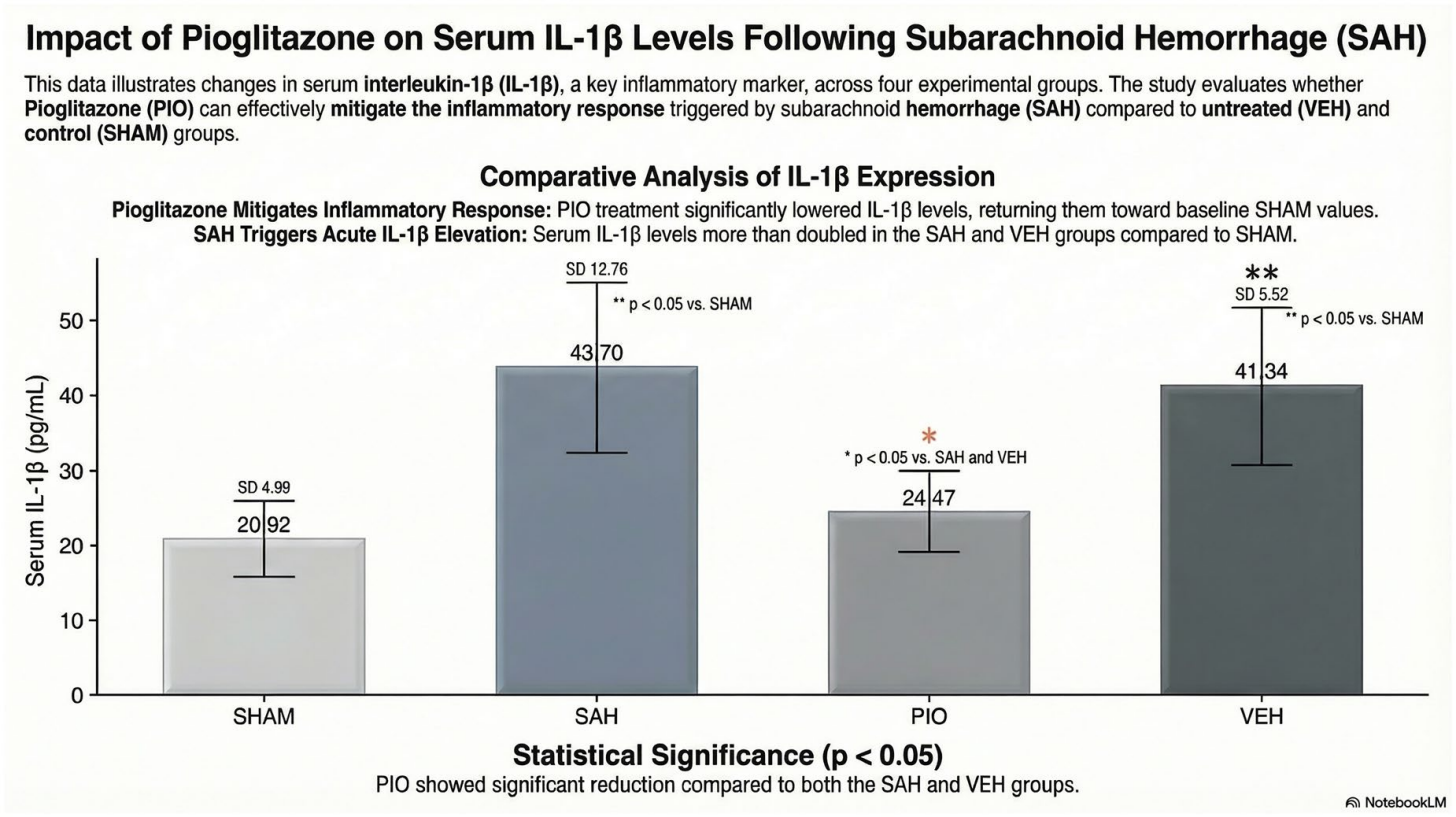
Serum IL-1β levels in experimental groups. Data are presented as mean ± SD; *p* < 0.05 was considered statistically significant. IL-1β levels in the PIO group were significantly lower than in SAH and VEH (*p* = 0.001 and *p* = 0.002, respectively).

Serum IL-1β levels were significantly elevated in the SAH group compared with the sham group (*p* < 0.001, Figure 3). In the SAH group, the mean IL-1β level was 43.70 ± 12.76 pg/mL. Pioglitazone administration significantly reduced IL-1β levels to 24.47 ± 5.52 pg/mL compared with the SAH group (*p* = 0.001). IL-1β levels in the PIO group were also significantly lower than those in the VEH group (*p* = 0.005). No significant difference was observed between the SAH and VEH groups (*p* = 0.564). Furthermore, IL-1β levels in the pioglitazone group were comparable to those in the sham group (*p* = 0.743).

### Histopathological findings

**Figure 4.**
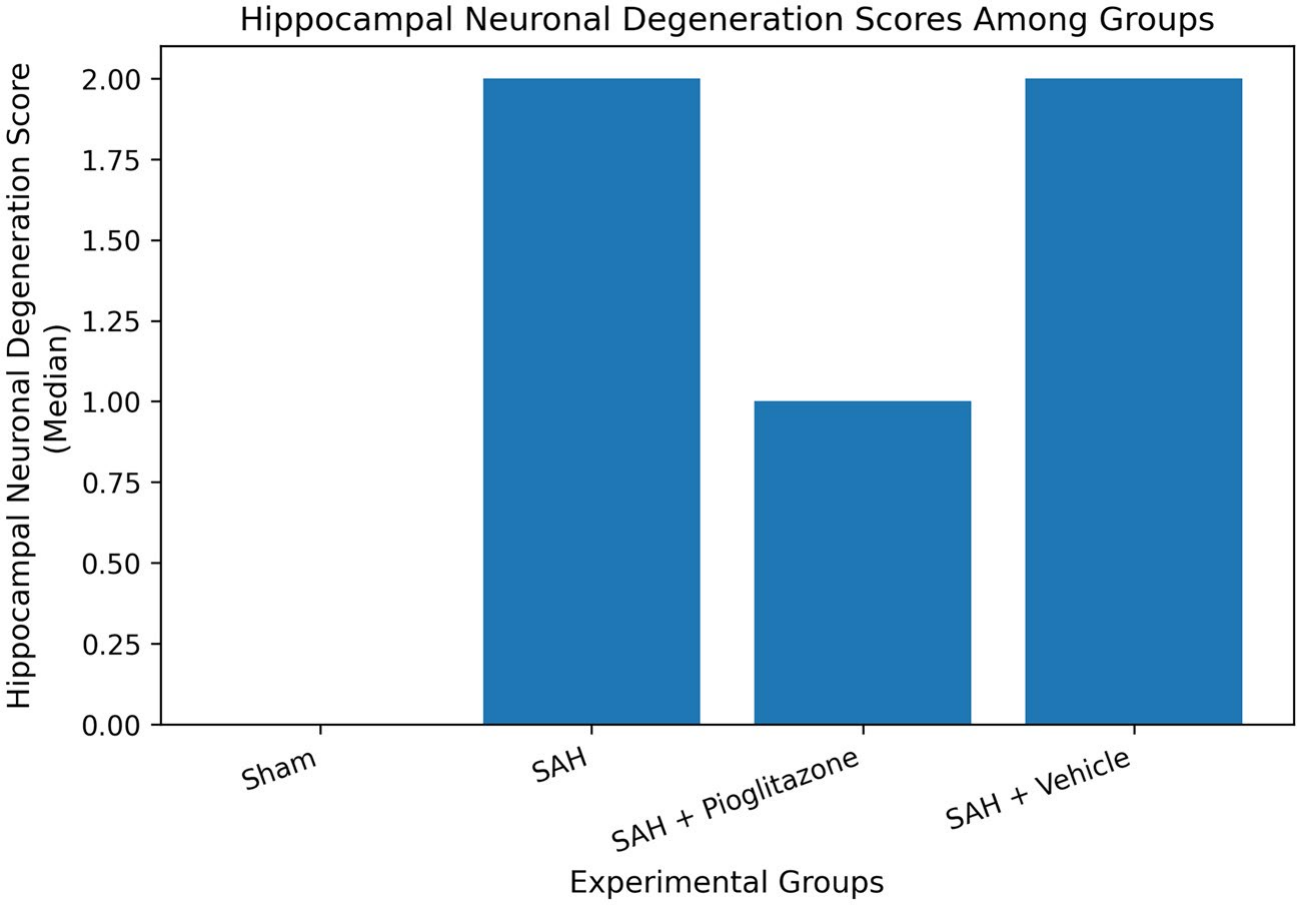
Semi-quantitative neuronal degeneration scores in the hippocampal CA1 region. Semi-quantitative neuronal degeneration scores in the hippocampal CA1 region. Quantification of hippocampal neuronal degeneration scores in all experimental groups (SHAM, SAH, PIO, VEH). Data are presented as median with interquartile range. Statistical analysis was performed using the Kruskal–Wallis test followed by Dunn’s post-hoc test. *p* < 0.05 compared to the SAH group.

**Figure 5.**
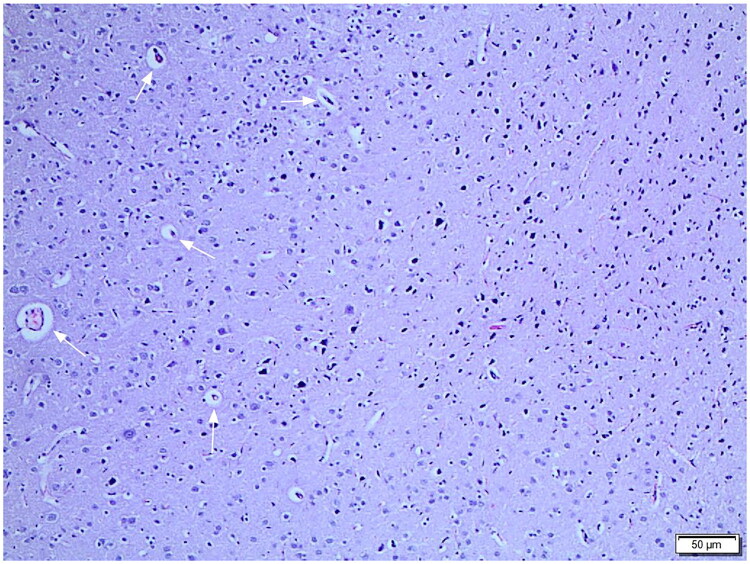
Neurodegeneration in the hippocampal CA1 after subarachnoid haemorrhage. Neurodegeneration in the Hippocampal CA1 After Subarachnoid Haemorrhage. Representative H&E-stained section of the hippocampal CA1 region in the SAH group. Arrows indicate neurons with pyknotic nuclei and eosinophilic cytoplasm. Scale bar = 50 µm.

Histopathological examination of hippocampal tissue samples revealed significant neuronal damage in groups with induced subarachnoid haemorrhage (Figure 4). Degenerative changes characterised by neuronal shrinkage, cytoplasmic eosinophilia, nuclear pyknosis, and localised cellular disarray were commonly observed in the SAH group **(**Figure 5). Similar findings were also detected in the VEH group, which received only a carrier substance, and no significant difference was observed between the two groups in terms of histopathological appearance (*p* > 0.05, Figure 4).

In the pioglitazone-treated group, the hippocampal neuronal architecture was largely preserved, and degenerative findings were significantly reduced. Neuronal shrinkage and pyknosis were more limited in the PIO group, and significant preservation of cellular integrity was noted.

According to semi-quantitative scoring results, the mean neuronal damage score was significantly higher in the SAH group. In the pioglitazone-treated group, histopathological damage scores were found to be significantly lower compared to the SAH group (*p* < 0.05). Similarly, damage scores in the PIO group were also significantly reduced compared to the VEH group (*p* < 0.05, Figure 4).

No significant difference was found between the SAH and VEH groups in terms of histopathological scores (*p* > 0.05). Although histopathological scores in the pioglitazone group were slightly higher than in the SHAM group, this difference was not statistically significant (*p* > 0.05). These findings indicate that pioglitazone treatment significantly reduces early hippocampal neuronal damage developing after subarachnoid haemorrhage.

In this study, pioglitazone administration resulted in a statistically significant reduction in serum IL-6, IL-1β, and MDA levels, which were significantly increased after subarachnoid haemorrhage, and in parallel with these biochemical changes, a significant improvement in hippocampal neuronal degeneration scores was observed in the pioglitazone group.

## Discussion

The present study demonstrates that a single intraperitoneal dose of pioglitazone hydrochloride (10 mg/kg) administered 1 h after SAH induction produces significant biochemical and histopathological improvement in early brain injury (EBI). Pioglitazone significantly reduced the SAH-induced elevations in serum IL-1β, IL-6, and MDA levels, and this biochemical improvement was accompanied by a marked reduction in hippocampal neuronal degeneration scores. Collectively, these findings support the concept that PPAR-γ activation modulates the inflammatory and oxidative pathways that are central to SAH pathophysiology. While our findings are consistent with the known roles of PPAR-γ in inflammation and oxidative stress, the precise mechanistic basis underlying the observed protective effects of pioglitazone, particularly regarding specific downstream molecular signalling pathways such as NF-κB and Nrf2, was not directly evaluated in this study and requires further investigation. In light of emerging research, future studies could also explore novel therapeutic strategies for SAH and the potential role of microRNAs as biomarkers in SAH [23,24].

An important strength of the present study is the use of a therapeutically relevant treatment paradigm. Although numerous studies have evaluated the neuroprotective effects of PPAR-γ agonists, many administered pioglitazone or related agents prophylactically or concurrently with haemorrhage [18,25]. In contrast, pioglitazone was administered 1 h after SAH induction in our model, demonstrating that the drug retains significant efficacy even after the pathological cascade has been initiated. This design more closely reflects the clinical timing of treatment initiation and enhances the translational relevance of our findings [26].

It is well established that post-SAH inflammation is a key driver of EBI. The dissemination of blood degradation products into the subarachnoid space triggers microglial activation, leading to increased production of proinflammatory cytokines such as IL-1β, IL-6, and TNF-α [6,15]. This process involves a complex interplay between peripheral immune cell recruitment and central microglial activation, contributing to a positive feedback loop that exacerbates neuroinflammation and secondary brain injury. In the present study, the significant reductions in serum IL-1β and IL-6 levels following pioglitazone treatment indicate suppression of the systemic—and potentially central—inflammatory response. Nevertheless, circulating cytokines may partially reflect systemic inflammatory activation rather than exclusively brain-specific processes; therefore, serum biomarker changes should be interpreted cautiously when attributing them solely to intracerebral inflammatory mechanisms. These results are consistent with prior reports demonstrating that PPAR-γ agonists attenuate cytokine production by modulating microglial activation in experimental brain injury models [15,27]. The suppression of these cytokines likely prevents the secondary cascade of blood-brain barrier disruption and further neuronal loss. It is important to note that despite pioglitazone treatment, IL-6 levels remained significantly higher than in the sham group. This partial effect suggests that while pioglitazone effectively modulates the inflammatory response, it may not completely normalise all inflammatory markers within the acute 24-hour window, possibly due to the complexity of SAH-induced inflammation or the single-dose regimen employed. Future studies could investigate the effects of different dosing strategies or longer treatment durations on the complete resolution of inflammatory markers.

Similarly, numerous studies have reported that anti-inflammatory interventions exert protective effects on EBI in experimental SAH models. For instance, Zhang et al. demonstrated that pioglitazone improved neurological outcomes by suppressing the inflammatory response in an experimental SAH model [18]. However, the literature also indicates that the magnitude of the anti-inflammatory effects of PPAR-γ agonists is not uniform across experimental settings [28]. This variability is likely attributable to differences in animal species, injury models, dosing regimens, routes of administration, and treatment timing [29]. In the present study, we speculate that the intraperitoneal route and early post-injury administration may have contributed to the more pronounced anti-inflammatory effects observed.

Oxidative stress represents another pivotal component of EBI following SAH. Haemoglobin degradation products and reperfusion processes promote excessive ROS generation, thereby triggering lipid peroxidation [7,30]. In the present study, pioglitazone treatment reduced serum MDA levels to values approaching those of the sham group, indicating a robust suppressive effect on oxidative stress. This observation is consistent with previous reports demonstrating that PPAR-γ agonists attenuate oxidative injury across various models of neurological damage [31,32]. Given the emerging role of ferroptosis in SAH, the marked reduction in MDA—a key marker of lipid peroxidation—suggests that pioglitazone may exert its neuroprotective effects by inhibiting iron-dependent cell death pathways.

Our histopathological findings further corroborate the biochemical data. Whereas the SAH group exhibited prominent neuronal degeneration, hippocampal neuronal architecture was largely preserved and degeneration scores were significantly reduced in the pioglitazone-treated group. These results suggest that the anti-inflammatory and antioxidant effects of pioglitazone translate into structural neuroprotection at the cellular level. Consistent with our findings, previous studies have reported that PPAR-γ agonists mitigate neuronal loss and preserve tissue integrity in experimental models of brain injury [33,34]. These findings align with recent clinical literature highlighting the dynamic changes in circulating biomarkers during the early phase of aneurysmal SAH [35].

However, the literature also emphasises that the histopathological improvement observed in the acute phase may not always directly correlate with long-term functional recovery [36]. Therefore, further studies are needed to evaluate how the histological protective effect we obtained will be reflected in long-term neurological outcomes. Long-term follow-up studies, especially those supported by behavioural tests and neurological scoring, will reveal the clinical potential of pioglitazone more clearly.

This study has shown that the therapeutic application of pioglitazone in the early period after the induction of SAH can provide significant biochemical and histopathological improvement. In this respect, the study differs from the existing literature, which is dominated by prophylactic or concomitant applications, and offers an approach closer to clinical practice. In the same experimental model, it was shown that the improvement in inflammatory (IL-1β, IL-6) and oxidative stress (MDA) markers parallels hippocampal neuronal protection. The demonstration that a single dose of pioglitazone can provide significant protective effects against early brain damage strengthens the potential translational value of PPAR-γ agonists in the treatment of SAH.

Therefore, the present findings should be interpreted as hypothesis-generating and provide a basis for further experimental and clinical studies investigating the potential role of PPAR-γ agonists in SAH. Although the present findings demonstrate promising biochemical and histopathological effects, caution should be exercised when extrapolating results from experimental animal models to clinical SAH populations. Future studies integrating molecular pathway analyses, behavioural assessments, and long-term outcome measurements will be essential to fully elucidate the neuroprotective potential of pioglitazone following SAH.

## Limitations

Our study has several limitations. First, only acute-phase biochemical and histopathological parameters were evaluated, and long-term functional outcomes were not assessed. This design choice was made to specifically investigate the early brain injury phase, which is critical for understanding acute pathophysiological mechanisms. However, we acknowledge that the lack of subacute or long-term functional assessments limits the translational relevance of our findings, and future longitudinal studies incorporating behavioural tests are warranted. In addition, the relatively small sample size used in this experimental model may limit statistical power and the generalisability of the findings. While our rodent model provides valuable insights into the acute phase of SAH, it inherently simplifies the complex and heterogeneous nature of human SAH. Future research could explore more complex animal models or incorporate patient-derived data to better mimic the clinical diversity of the human condition. In addition, the relatively small sample size used in this experimental model, despite being supported by a priori power analysis, may limit statistical power and the generalisability of the findings. Furthermore, a specific antagonist was not used to confirm the PPAR-γ–mediated mechanism of pioglitazone. However, the observed effects are highly consistent with established PPAR-γ signalling pathways, and the primary aim was to demonstrate the therapeutic potential of pioglitazone in the acute phase.

Previous studies have reported that pioglitazone may exert dose- and time-dependent effects [21,37]. In this context, although the single-dose, single-timepoint protocol used in our study yielded promising results, further studies comparing different dosing regimens and treatment timings are needed to determine the optimal therapeutic strategy. Furthermore, while we focused on serum markers, future studies incorporating cerebrospinal fluid (CSF) analysis and more detailed molecular markers of ferroptosis would provide a more comprehensive understanding of the central effects. Future studies incorporating multiple time points and functional neurological assessments would provide a more comprehensive understanding of the therapeutic potential of pioglitazone following SAH.

Overall, these findings clarify the effects of pioglitazone on the inflammatory and oxidative processes involved in the pathophysiology of early brain injury after SAH and provide additional experimental evidence supporting the therapeutic potential of PPAR-γ agonists in this field.

## Conclusion

This study demonstrates that a single dose of pioglitazone administered 1 h after SAH significantly reduces hippocampal neuronal damage by suppressing systemic inflammation and lipid peroxidation. Our findings indicate that PPAR-γ activation provides meaningful biochemical and histopathological improvement during the early phase of brain injury. The fact that pioglitazone is a well-established agent in clinical use enhances the translational relevance of this approach and suggests a promising pharmacological strategy for acute neurological conditions with limited therapeutic options, such as SAH. Notably, the marked reduction in serum MDA levels supports the protective role of the antioxidant and anti-inflammatory effects of pioglitazone in EBI and provides a rationale for further mechanistic and translational studies.

## Data Availability

Data from this study are available from the corresponding author upon reasonable requests.
